# Specificity protein 1/microRNA-92b forms a feedback loop promoting the migration and invasion of head and neck squamous cell carcinoma

**DOI:** 10.1080/21655979.2021.2008698

**Published:** 2021-12-14

**Authors:** Pai Pang, Hui Fang, Hong Wu, Song Wang, Minda Liu, Shan Jin, Zhongzheng Qi, Zhenning Li, Fayu Liu, Changfu Sun

**Affiliations:** Department of Oral and Maxillofacial Surgery, School and Hospital of Stomatology, China Medical University, Liaoning Provincial Key Laboratory of Oral Diseases, No.117, Nanjing Bei Street, Heping District, Shenyang, Liaoning, 110002, People’s Republic of China

**Keywords:** Head and neck squamous cell carcinoma, microRNA-92b, specificity protein 1, feedback loop, migration and invasion

## Abstract

In this study we report a novel specificity protein 1 (SP1)/microRNA-92b (miR-92b) feedback loop regulating the migration and invasion of head and neck squamous cell carcinoma (HNSCC). Microarray and real-time Polymerase Chain Reaction (PCR) were used to detect gene expression in HNSCC tissues and cell lines. Transwell migration, invasion, wound healing and cell counting kit – 8 (CCK-8) cell assays were used to compare cell migration, invasion and proliferation abilities. Chromatin Immunoprecipitation (ChIP) assays were used to detect SP1 binding to the miR-92b promoter. Western blot was used to detect protein levels. An in vivo tumorigenesis experiment was used to evaluate the effect of SP1 knockdown on tumor growth and protein levels were evaluated by immunohistochemistry. We found that the miR-92b expression level was elevated in HNSCC primary focus tissue compared with adjacent normal tissue, and a higher level of miR-92b was related to a higher clinical stage and worse prognosis of HNSCC patients. MiR-92b and SP1 mutually promoted each expression and cooperatively facilitated the migration, invasion and proliferation of HNSCC cells. A decreased level of SP1/miR-92b resulted in a restraint of in vivo tumor growth. In conclusion, our results suggest that the SP1/miR-92b feedback loop generally promotes HNSCC invasion and metastasis, thus presenting a possible therapeutic target in the treatment of HNSCC patients.

## Introduction

Head and neck squamous cell carcinoma (HNSCC) is one of the most vicious diseases in the head and neck region and develops from the epithelium of the oral cavity, pharynx, larynx and so on [1]. In 2020, HNSCC accounted for more than 931,000 new cases and over 467,000 deaths globally according to global cancer statistics [2]. Although there has been a rapid growth of scientific researche concerning HNSCC in recent years, the features of locoregional invasion and metastasis to cervical lymph nodes render its 5 year survival rate at approximately 60% [3]. It is estimated that nearly 50% of HNSCC patients have recurrence and cervical lymph node metastasis within 5 years after diagnosis. For these patients, the reported 5-year overall survival rate is as low as 43.8% [4,5]. Therefore, a better understanding of the underlying biological mechanism of HNSCC progression and metastasis is desirable.

Recently the role of the regulatory network between transcription factors (TFs) and microRNAs in genetic disorders and systemic diseases has gradually aroused additional attention [6]. Specificity protein 1 (SP1) is a zinc finger transcription factor that binds to GC-rich motifs of many promoters and participates in the modulation of cell proliferation and survival, evading growth suppression, resisting cell death, promoting angiogenesis and activating cancer cell invasion and metastasis by interacting with important biological molecules including microRNAs [7,8]. It has been reported that SP1 can regulate microRNA expression at the transcriptional level, while in several human cancers SP1 acts as a downstream target molecule and is posttranscriptionally modulated by miRNAs [9–11].

MicroRNAs are small (19–25nt), noncoding RNAs that regulate downstream gene expression at a posttranscriptional level by binding to the 3ʹ untranslated regions (UTRs) of target mRNA transcripts and causing translational repression or degradation [12]. Emerging evidence has illustrated the promising roles of microRNAs as biomarkers for diagnosing diseases and predicting the prognosis of cancer patients [13]. MicroRNA-92b (miR-92b) a the well-studied miRNA that has been reported to play a critical role in many human neoplastic diseases including breast cancer [14,15], lung cancer [16,17], esophageal cancer [18], and gastric cancer [19]. In addition to being regulated by TFs, miRNAs can sometimes form a regulatory loop with these TFs that participates in the process of cancer development and progression [20,21].

Although some studies have shown that miR-92b may be related to the proliferation and chemotherapy response of oral squamous cell carcinoma (OSCC) [22,23], its specific biological role and molecular mechanism in the invasion and migration of HNSCC are still unclear. In this regard, we hypothesized that miR-92b plays an important role in the development of HNSCC, and studied whether transcription factor SP1 promotes the invasion and migration of head and neck squamous cell carcinoma cells through the interaction with miR-92b, supporting a novel feed-forward loop with a significantly important role in the progression of HNSCC.

## Methods

### Clinical specimens

This research was approved by the ethics committee of the School of Stomatology of China Medical University and performed in accordance with the ethical standards of the Declaration of Helsinki as outlined in 1975, revised in 2008. Paired primary focus and adjacent nontumor tissues were obtained from 15 HNSCC patients treated between May 2014 and April 2015 at the Department of Oral and Maxillofacial Surgery, China Medical University. All cancer and adjacent control tissue samples were collected by experienced surgeons (ML, SJ and ZQ) and rechecked and confirmed by pathologists. Written informed consent was obtained from all participants. The clinical stages of patients were classified according to the TNM classification of malignant tumors from Union for International Cancer Control (UICC, 8th Edition). All tumor samples contained over 85% cancerous tissue before RNA extraction.

### Cell lines and cell transfection

HNSCC cell lines PCI-4A/4B and PCI-37A/37B were cultured at 37°C in a humidified atmosphere of 5% CO_2_ in Dulbecco’s modified Eagle’s medium (DMEM; Invitrogen, Carlsbad, CA, USA) supplemented with 10% fetal bovine serum (FBS; Gibco, Carlsbad, CA, USA), 100 U/ml penicillin G and 100 U/ml streptomycin [24]. The RNA oligonucleotide miR-92b mimics, inhibitor, SP1 siRNA, and negative control were synthesized by GenePharma (2603, GenePharma, Shanghai, China). MiR-92b inhibitor and negative control plasmid were purchased from GeneChem (PMDE249003218, GeneChem, Shanghai, China). The transfection agent Lipofectamine 2000 (11668500, Invitrogen) was used according to the manufacturer’s protocol [25]. Cells were harvested 24 hours after transfection for cell migration, invasion, proliferation and wound healing assays, 48 hours for RNA isolation and 72 hours for Western blot analysis. The sequences of siRNA: miR-92b mimics 5'-uauugcacucgucccggccuccAGGCCGGGACGAGUGCAAUAUU-3'; miR-92b mimics negative control (NC) 5'-uucuccgaacgugucacguttACGUGACACGUUCGGAGAATT-3'; miR-92b inhibitor 5'-GGAGGCCGGGACGAGUGCAAUA-3'; miR-92b inhibitor NC 5'-CAGUACUUUUGUGUAGUACAA-3'; SP1 siRNA 5'-ccagcaacaugggaauuauttAUAAUUCCCAUGUUGCUGGTT-3'; SP1 siRNA NC 5'-uucuccgaacgugucacguttACGUGACACGUUCGGAGAATT-3'; The sequences of shRNA: miR-92b inhibitor 5'-gatccGGAGGCCGGGACGAGTGCAATAttttt-3'; miR-92b inhibitor NC 5'-agctaaaaaTATTGCACTCGTCCCGGCCTCCg-3'.

### Quantitative real-time reverse-transcription polymerase chain reaction assay (real-time qRT-PCR)

Total RNA was isolated from HNSCC cell lines and tissue samples following the manufacturer’s instructions for RNAiso Plus (9108, Takara, Beijing, China). The experiments were performed with an ABI PRISM 7500 Real-Time PCR Reaction System (USA) following the instructions of the TB Green^TM^ Premix Ex Taq^TM^ II Kit (820A, Takara, Beijing, China). The primer sequences used were as follows: U6 forward 5'-CTCGCTTCGGCAGCACA-3'; U6 reverse 5'-AACGCTTCACGAATTTGCGT-3'; miR-92b forward 5'-CGCCGCTAAAGTGCTTATAGTG-3'9;; miR-92b reverse 5'-GTGCAGGGTCCGAGGTATTC-3'; GAPDH forward 5'-GAAGGTGAAGGTCGGAGTC-3'; GAPDH reverse 5'-GAAGATGGTGATGGGATTTC-3'; SP1 forward 5'-TGCAGCAGAATTGAGTCACC-3'; SP1 reverse 5'-CACAACATACTGCCCACCAG-3'. The expression levels of these targets were defined from the threshold cycle (Ct), and calculated by the 2^−ΔΔCt^ method after normalization to the level of reference genes [26]. All experiments were performed at least three times in triplicate.

### Microarray

Detailed information is provided in supplementary material S1 [27].

### Analysis of cell proliferation, migration, and invasion

Transwell migration assays and monolayer wound healing assays were used to evaluate cell migration. A Matrigel invasion assay was used to detect cell invasive ability [24]. Cell counting kit – 8 (CCK-8) assay was used to evaluate cell proliferation [28]. Details are provided in supplementary material S2.

### Bioinformatics analysis

Gene expression data and clinical information of HNSCC patients were downloaded from The Cancer Genome Atlas (TCGA) – HNSC database (https://portal.gdc.cancer.gov/). Finally gene expression data of 523 HNSCC patients were extracted and analyzed. Analysis of miR-92b and SP1 expression was performed via R package ‘edgeR” (R Foundation for Statistical Computing, Vienna, Austria) [29].

### Western blot analysis

Cells were harvested and lyzed in Radio-Immunoprecipitation Assay (RIPA) lysis buffer, and centrifuged at 4°C 12,000 g for 10 min. The concentration of each protein sample were measured using the BCA protein concentration assay kit (Wanleibio, Shenyang, China). Proteins (30 μg) extracted from cells were loaded and separated by 8% sodium dodecyl sulfate-polyacrylamide gel electrophoresis (SDS-PAGE) and then transferred to nitrocellulose membranes. The membranes were blocked with 5% nonfat milk and incubated with anti-SP1 (1:1000, 9389, Cell Signaling Technology, MA, USA) in 5% milk/PBS buffer at 4°C overnight, followed by incubation with goat anti-rabbit secondary antibody (A23720, Abbkine, Wuhan, China) conjugated with DyLight fluorescent 680. After extensive washing with PBST buffer, the protein band was visualized under an Odyssey CLx imaging system (LI-COR, Inc., NE, USA) [30].

### Chromatin immunoprecipitation (ChIP) assay

PCI-4A cells were first fixed with 1% formaldehyde for 10 minutes at room temperature to covalently crosslink proteins to DNA. Then, the cells were lysed and sonicated to generate DNA fragments at 500–1000 base pairs in length followed by pre-clearing with Salmon Sperm DNA/Protein A Agarose-50% Slurry at 4°C for 2 hours. After, the samples were subjected to an immunoselection process and incubated with SP1 immunoprecipitating antibody (1:50, 9389, Cell Signaling Technology) at 4°C overnight. The immunocomplexes were recovered by protein A beads at 4°C for 2 hours and eluted with wash buffer. The crosslinks were reversed with 5 M NaCl at 65°C overnight. DNA samples were purified and SP1 enrichment at the miR-92b promoter region binding site was measured by PCR. PCR primers: site1 forward and experimental groups are listed in Table S1 and supplementary material S3 [31].

### Lentivector construction and cell infection

The lentivector Psico was purchased from Addgene (#11,578, Addgene, MA, USA). After being double digested with HpaI/XhoI, the vector was connected with synthetic SP1 knockdown or NC sequences. After being screened through sequencing, positive lentivectors carrying the SP1 knockdown sequence or NC were selected for cell infection. PCI-37B cells cultivated in 6-well plates at a confluence of 70% were cocultured with lentivirus suspension (SP1 knockdown 1:100; NC 1:200) for 48 hours. Then the cells were cultured with complete culture medium with 15 µg/ml puromycin for one week to harvest strains that had stably low levels of SP1 expression [30].

### In vivo tumorigenesis in nude mice

For subcutaneous injection, 1 × 10^7^ blank control, SP1 control and SP1 knockdown PCI-37B cells in 200 µL DMEM were subcutaneously injected into the axillary tissue of BALB/c nude mice with 5 mice in each group. The length and width of tumor were measured. Twenty-five days after injection, the mice were sacrificed and the tumors were excised and weighed [30]. This study was conducted in accordance with Interdisciplinary Principles and Guidelines for the Use of Animals in Research, Testing, and Education by the New York Academy of Sciences, Ad Hoc Animal Research Committee and the experimental protocols were approved by the ethics review committee for animal experimentation of China Medical University.

### Immunohistochemical staining

For immunohistochemical staining, the paraffin-embedded sections were incubated with primary antibody against SP1 (1:200, 9389, Cell Signaling Technology) at 4°C overnight. Then, the cells were incubated in biotinylated goat anti-rabbit IgG at 37°C for 30 min, followed by incubation with herseradish peroxidase (HRP)-labeled avidin. After visualization with diaminobenzidine (DAB), the sections were counterstained with hematoxylin and observed at 400x. The expression of SP1 protein was semiquantitatively measured by using Image-Pro Plus 6.0 (Media Cybernetics, Inc., MD, USA) [32].

### Statistical analysis

Data are expressed as the mean ± standard deviation (SD) from at least three independent experiments. Student’s t test was used to compare gene expression, migrating or invading cells, healing percentage, cell viability and tumor volume. The Mann-Whitney U test was used to evaluate the associations between miR-92b expression and the clinical characteristics of HNSCC patients. In the prognosis analysis of overall survival (OS), Kaplan–Meier curves were used and tested by the log-rank test. Univariate and multivariate Cox proportional hazard regression analyses were used to identify risk factors and their hazard ratios (HRs) and 95% confidence intervals (CIs) related to HNSCC patient death. Statistical analysis was performed with SPSS 21.0 (SPSS, Inc., Chicago, IL, USA). A p value of less than 0.05 was considered statistically significant.

## Results

In this study, we aimed to investigate the role of miR-92b and transcription factor SP1 in HNSCC invasion and metastasis. Through a series of in vitro assays, we found that miR-92b and Sp1 formed a positive feedback regulatory loop that promoted the migration, invasion and proliferation of HNSCC, and promoted tumor growth in vivo.

### MiR-92b is involved in the migration and invasion of HNSCC cells

**Figure 1.**
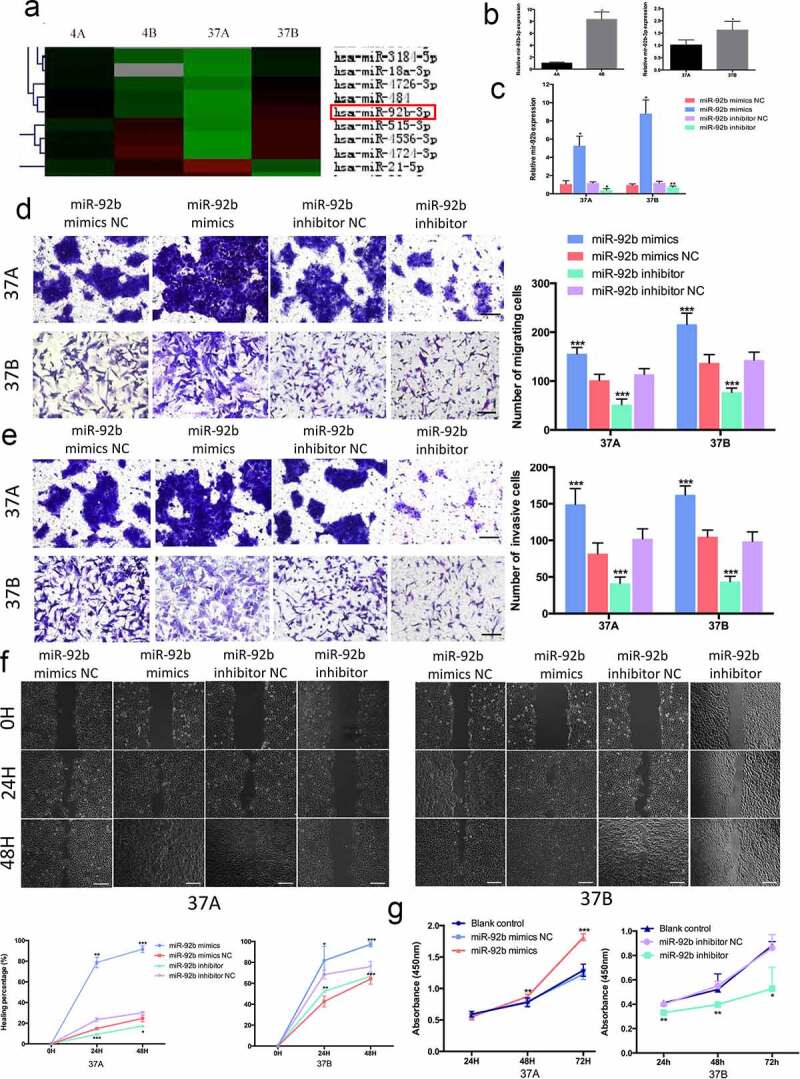
MiR-92b promotes HNSCC cell migration, invasion and proliferation. (a) Microarray results show that miR-92b expression levels are higher in PCI-4B and PCI-37B cells than in PCI-4A and PCI-37A cells; (b) MiR-92b expression in HNSCC cells verified by real-time qPCR; (c) MiR-92b expression levels in cells transfected with miR-92b mimics, inhibitor and NC; (d) Mir-92b promotes migratory ability of PCI-37A/B cells, scale bar 50 µm; (e) Mir-92b promotes invasive ability of PCI-37A/B cells, scale bar 50 µm; (f) Wound healing images of PCI-37A/B cells transfected with miR-92b mimics, inhibitor and NC at 0, 24 and 48 hours after scratch, scale bar 100 µm; (g) In CCK8 assay, an increase in miR-92b expression in PCI-37A cells significantly promotes cell viability at 48 and 72 hours after transfection, and a decrease in miR-92b expression in PCI-37B cells inhibits proliferation at 24, 48 and 72 hours after transfection. All experiments were repeated at least three times. *p < 0.05 compared with the NC group; **p < 0.01 compared with the NC group; ***p <0.001 compared with the NC group

We first identified miR-92b as one of the most differentially expressed microRNAs in two pairs of cell lines PCI-4A/B and PCI-37A/B by microarray (Figure 1(a)). PCI-4A/B and PCI-37A/B are cell lines constructed from two different HNSCC patients. PCI-4A and PCI-37A were constructed from primary HNSCC focus and PCI-4B and PCI-37B were constructed from corresponding metastases with higher migratory and invasive abilities [33,34]. Real-time RT-PCR used to validate this difference indicated that the expression level of miR-92b in PCI-4B cells was 8.3 times as high as that in PCI-4A cells and that in PCI-37B cells was 1.6 times as high as that in PCI-37A cells (Figure 1(b)).

Then we transfected RNA oligo miR-92b mimics or inhibitor to PCI-37A cells into exogenously regulate its expression (Figure 1(c)). Transwell migration assays and Martrigel invasion assays showed that an ectopically increased level of miR-92b could increase the migration and invasion ability of PCI-37A cells, while a reduced level of miR-92b led to a significant decrease in the number of migrating or invasive cells (Figure 1(d,e)). Additionally, in the wound-healing assay high levels of miR-92b accelerated wound closure by 63.97% at 24 h and by 66.96% at 48 h after transfection in comparison with negative control group. Additionally, an apparent reduced repopulation area was observed at 24 h and 48 h after transfection in the miR-92b knockdown group (Figure 1(f)). Similar results were obtained from the experiments performed with PCI-37B cells.

In addition, we elevated miR-92b levels in PCI-37A cells and downregulated its expression in PCI-37B cells, and then evaluated the cell proliferation abilities by using a CCK-8 assay. The results demonstrated that compared with the corresponding control groups, cell viability was significantly increased in the 37A miR-92b mimic group at 72 h after transfection, and clearly restrained at 48 h and 72 h after cell transfection in the 37B miR-92b inhibitor group (Figure 1(g)).

### MiR-92b is a marker of metastasis and prognosis for HNSCC

**Figure 2.**
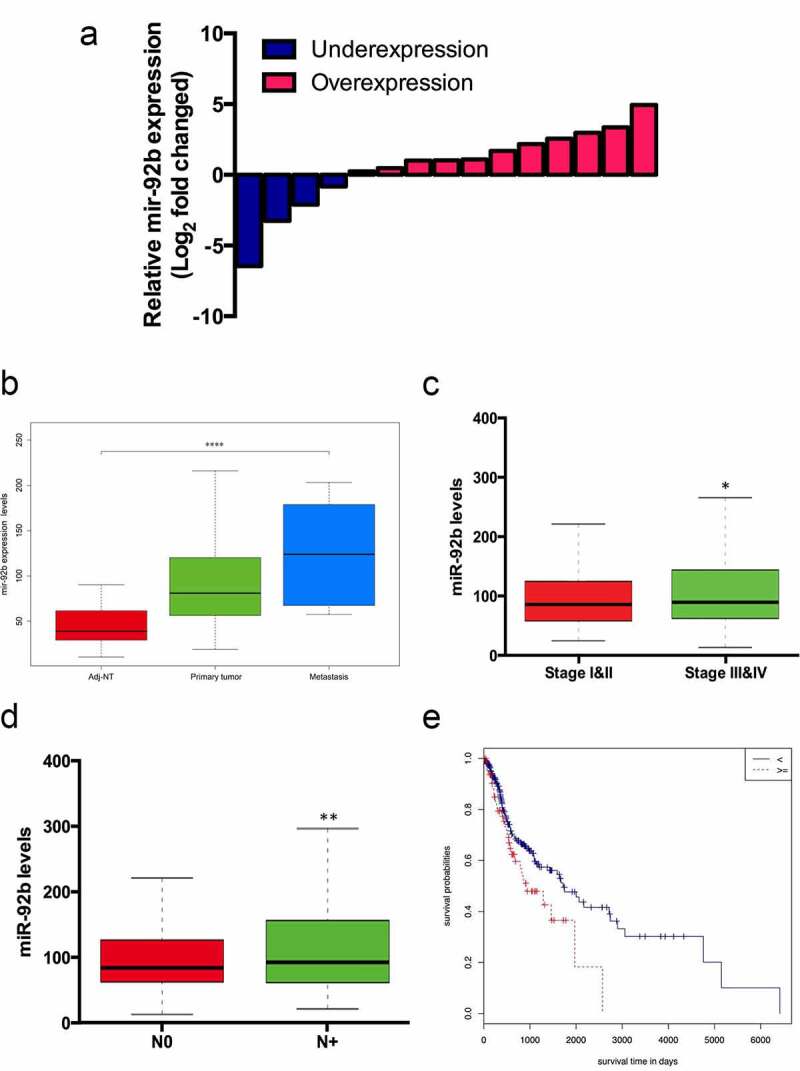
MiR-92b levels are associated with HNSCC progression and metastasis. (a) MiR-92b expression levels in HNSCC patient specimens. (b) Expression levels of miR-92b in adjacent normal tissue, primary tumor and metastatic focus in HNSCC patients from TCGA-HNSC dataset, p<0.0001; (c) MiR-92b expression in stage I&II versus stage III&IV HNSCC patients, p=0.012; (d) MiR-92b expression in N0 versus N+ HNSCC patients, p=0.004; (e) Kaplan-Meier analysis of survival probabilities of HNSCC patients with high and low miR-92b levels in primary tumors, p=0.031. All experiments were repeated at least three times. *p<0.05 compared with the NC group p<0.05; **p<0.01 compared with the NC group; ****p<0.0001 compared with the NC group

To further investigate the role of miR-92b in HNSCC progression, we detected miR-92b expression in 15 paired HNSCC primary focus and adjacent normal tissues (Table S2). We found that miR-92b was elevated in cancerous tissue in 11 (73.33%) patients, and in 81.82% of them miR-92b levels were upregulated over 2 fold (Figure 2(a)). Then we extracted and analyzed the gene expression data of 523 HNSCC patients from the TCGA database. The results indicated that miR-92b expression levels were significantly higher in tumors than in adjacent noncancerous tissues, and further elevated in metastatic lesions (Figure 2(b)). MiR-92b was upregulated in stage III & IV HNSCC patients compared with stage I & II patients (Figure 2(c)). Additionally patients with lymph node metastasis expressed higher levels of miR-92b than N0 patients (Figure 2(d)). By using receiver-operating characteristic (ROC) analyses, we divided the patients into miR-92b-high and miR-92b-low groups. Based on Kaplan–Meier analysis, higher expression of miR-92b was significantly correlated with a lower survival rate (Figure 2(e)).

By using the Mann–Whitney test we found that miR-92b levels were related to patients’ lymph node metastasis status (Table 1). Cox proportional hazard regression analysis revealed that T stage, N stage and high miR-92b levels are independent risk factors related to the death of HNSCC patients (Table 2). Further multivariate analysis indicated that elevated miR-92b expression was associated with higher risk of death in HNSCC patients (HR = 2.505; 95% CI:1.027–6.106; P = 0.043).

**Table 1.** Correlation of miR-92b expression and clinical characteristics of HNSCC patients

Characteristics	Number of patients	miR-92b expression level	P-value
Gender			0.014*
Male	360	108.90 ± 77.87	
Female	132	87.60 ± 57.02	
Age			0.387
<60	222	108.41 ± 79.83	
≥60	251	98.21 ± 67.40	
Alcohol history			0.221
Yes	329	109.17 ± 77.32	
No	156	92.50 ± 64.28	
Clinical stage			0.802
I&II	96	104.45 ± 67.84	
III&IV	326	100.29 ± 74.94	
T stage			0.27
T1/2	171	98.41 ± 67.80	
T3/4	262	101.76 ± 73.88	
N stage			0.019*
N1/2	169	93.80 ± 67.75	
N3/4	253	105.16 ± 78.54

**Table 2.** Univariate and multivariate analysis of miR-92b prognosis

	Univariate analysis			Multivariate analysis		
Characteristics	HR	95% CI	P value	HR	95% CI	P value
Age (<60,≥60)	0.819	0.641–1.046	0.11			
Gender (male,female)	1.406	1.053–1.876	0.021*	1.411	1.055–1.887	0.02*
Alcohol use (YES,NO)	1.041	0.801–1.352	0.766			
Clinical stage (I/II,III/IV)	1.185	0.945–1.485	0.141			
T stage (T1/2,T3/4)	1.321	1.078–1.618	0.007*	1.223	0.993–1.508	0.059
N stage (N0,N+)	1.337	1.103–1.619	0.003*	1.27	1.039–1.552	0.02*
miR-92b expression (Low/high)	2.661	1.094–6.472	0.031*	2.505	1.027–6.106	0.043*

### SP1/miR-92b forms a positive feedback loop in HNSCC migration and invasion

**Figure 3.**
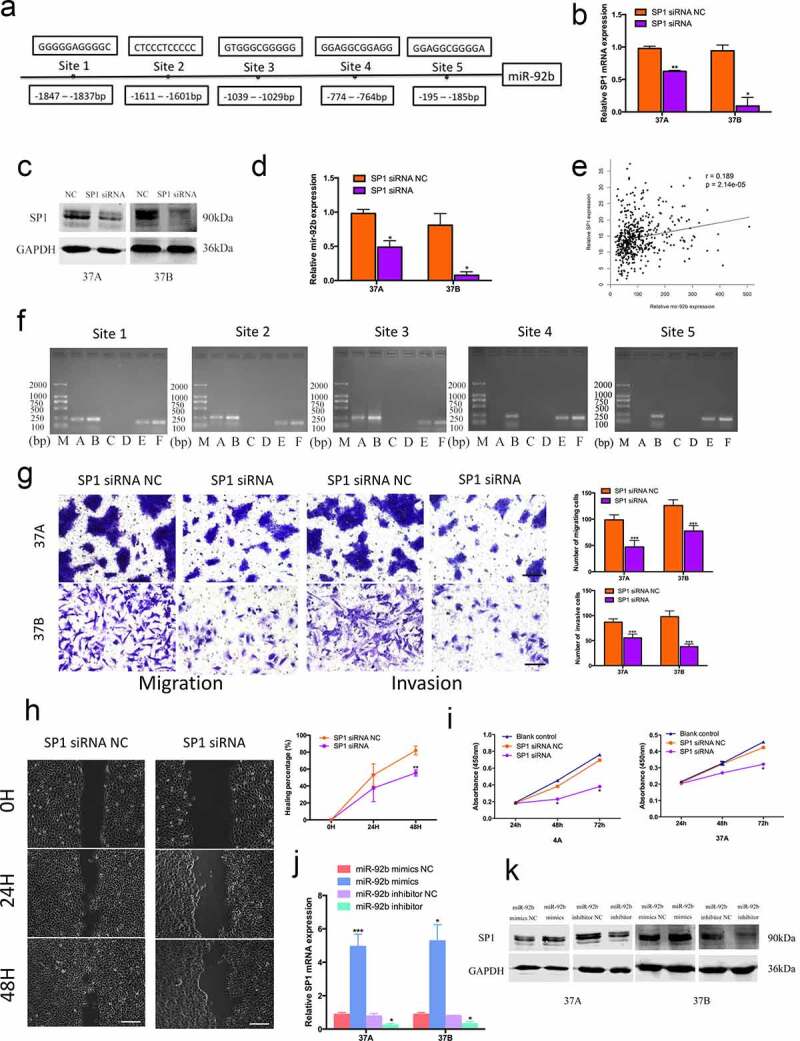
SP1 and miR-92b form a positive feedback loop in HNSCC cells. (a) Five possible SP1 binding sites on miR-92b promoter region identified via bioinformatics analysis; (b) SP1 mRNA expression levels in SP1 siRNA and NC groups of PCI-37A/B cells; (c) SP1 protein levels in SP1 siRNA and NC groups of PCI-37A/B cells; (d) Downregulating SP1 expression causes a decrease of miR-92b levels; (e) The SP1 expression levels increase with the increase of miR-92b levels in HNSCC patients’ specimens in TCGA-HNSC dataset, p<0.0001; (f) ChIP assay verifies that SP1 binds to the predicted site 1-3 in miR-92b promoter: (M) marker, (A) experiment group, (B) RT-Input1, (C) negative control group, (D) PCR negative control, (E) positive control group, (F) RT-Input2; (g) Downregulating SP1 expression results in a decrease of cell migratory and invasive abilities in PCI-37A/B cells, scale bar 50 µm; (h) Wound healing images of PCI-37A/B cells transfected with SP1 siRNA and control groups at 0, 24 and 48 hours after scratch, scale bar 100 µm; (i) Proliferation of PCI-4A/37A cells transfected with SP1 siRNA or control groups at 24, 48 and 72 hours after transfection; (j) SP1 mRNA expression levels in PCI-37A/B cells after transfected with miR-92b mimics and inhibitor; (k) SP1 protein levels in PCI-37A/B cells transfected with miR-92b mimics, inhibitor and control groups. All experiments were repeatedat least three times. *p<0.05 compared with the NC group; **p<0.01 compared with the NC group; ***p<0.001 compared with the NC group

By using TRANSFAC®7.0, CHIPBase, JASPAR, PROMO and P – MATCH bioinformatic software we found 5 possible SP1 binding motifs in the miR-92b promoter region (Figure 3(a)). After transfected with SP1 siRNA (Figure 3(b,c)), PCI-37A/B cells showed a clearly reduction in miR-92b expression (Figure 3(d)). By analyzing gene expression data from TCGA database we found that the SP1 expression level increased with increasing miR-92b levels (Figure 3(e)). We performed ChIP assays with PCI-4A cells to confirm the binding of the 5 sites in the miR-92b promoter region. The results indicated that there was a significant enrichment of the SP1 protein at the first 3 binding sites, whereas no clear reaction was found at site 4 and 5 (Figure 3(f)).

After knockdown with SP1 expression, PCI-37A/B cells showed a significant decrease in migration and invasion (Figure 3(g)). A wound healing assay in PCI-37A cells revealed that the healing percentage of the SP1 siRNA group was decreased by 15.47% at 24 h and by 26.51% at 48 h in comparison with the negative control group (Figure 3(h)). The cell viability of the SP1 siRNA group was also lower than that of the control group at 48 h and 72 h after transfection (Figure 3(i)). To further investigate whether miR-92b conversely affected SP1 function, we transfected miR-92b mimics and inhibitor into HNSCC cells, and found a parallel change in both SP1 mRNA and protein expression (Figure 3(j,k)). These results indicated that SP1 and miR-92b form a positive feedback loop in HNSCC and synergistically promote cancer cell metastasis and invasion.

**Figure 4.**
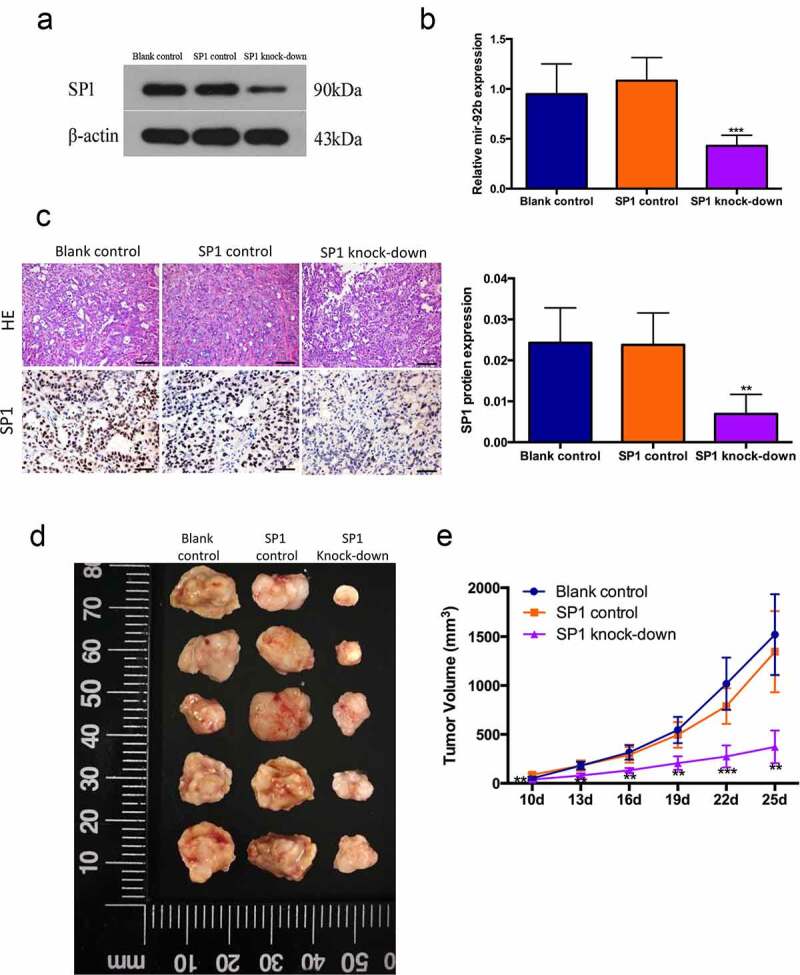
SP1/miR-92b feedback loop affects tumor growth in vivo. (a) SP1 protein levels in blank control, SP1 control and SP1knockdown group cells; (b) MiR-92b levels in xenograft tumors of blank control, SP1 control and SP1 knockdown groups; (c) HE staining and immunohistochemistry staining of SP1 in xenograft tumors of blank control, SP1 control and SP1knockdown groups, scale bar 50um. (d) Images of xenograft tumor of blank control, SP1 control and SP1 knock-down groups; (e) In vivo tumor growth of the blank control, SP1 control and SP1 knockdown groups; All experiments were repeated at least three times. **Compared with the NC group p<0.01; ***Compared with the NC group p<0.001

Then we verified this regulation in vivo by using a xenograft model in nude mice with PCI-37B cells transfected with lentiviral vector carrying SP1 knockdown sequences (Figure 4(a)). In comparison with the blank and control groups, the SP1 knockdown group had a decreased levels of miR-92b and SP1 expression, suggesting that the SP1/miR-92b positive feedback loop was generally knocked down (Figure 4(b,c)). The SP1 knockdown group also showed a significantly reduced tumor growth speed and volume (Figure 4(d,e)). We hereby speculated that activation of the SP1/miR-92b loop generally promotes tumor progression in vivo.

## Discussion

In this study we presented data about three major points: First, miR-92b overexpression contributes to high clinical stages and poor prognosis of HNSCC patients, second, miR-92b is transcriptionally activated by SP1 and simultaneously promotes its expression, and finally, the SP1/miR-92b positive feedback loop facilitates HNSCC cell migration and invasion in vitro and tumor growth in vivo.

SP1 is well-characterized transcription factor that can regulate many classic molecules and signaling pathways including epidermal growth factor receptor (EGFR), p53 and vascular endothelial growth factor (VEGF) to participate in cell survival, apoptosis, migration and many other biological processes that are crucial in oncogenesis and cancer development [35]. In addition to genomic control, recent studies have revealed the modification of SP1 mRNA from epigenomic factors including microRNAs. Several microRNA precursors have been reported to repress ZBTB4 and ZBTB10, which can competitively bind to GC-rich sequences in the promoter region and displace the SP1 protein to weaken its function [36,37]. Additionally microRNAs including miR-223 and miR-31-5p could regulate SP1 levels posttranscriptionally process by binding to its 3ʹ-UTR region [38–40]. In particular, SP1 can form an autoregulatory feedback loop with certain microRNAs and participate in the regulation of tumor progression as a whole. Kong et al. reported that a miR-22/Sp1/c-Myc network affected the invasive, metastatic and proliferative capacities of breast cancer cells by regulating CD147 expression [41]. Fulciniti also found that in multiple myeloma cells the miR23b/SP1/c-myc feed-forward loop could activate caspase-3 thus promoting cell proliferation [42]. From these studies we suggest that a typical TF – microRNA regulatory network often involves a miRNA that acts as a tumor suppressor and a transcription factor that is simultaneously downregulating and downregulated by this miRNA. However, in this study we identified SP1/miR-92b as a positive feedback loop that promotes the migration and invasion of HNSCC cells. As direct microRNA-mediated RNA activation (RNAa) has only been reported for several specific microRNA-gene pairs [43–45], and we did not find any miR-92b incorporable sequence in the SP1 promoter region yet, we provisionally suppose that miR-92b could upregulate SP1 levels in an indirect way by interacting with other intermediate elements.

In the process of preparing this manuscript, we found that chemokine receptor 7 (CCR7) is a likely common downstream target molecule of SP1 and miR-92b. CCR7 is a potent G-protein-coupled receptor (GPCR) that performs its biological function by interacting with its two ligands, C-C Motif Chemokine Ligand 19 (CCL19) and CCL21 [46]. Previous stdies have shown that CCR7 can promote HNSCC metastasis and invasion through the Phoshatidylinositol 3 kinase (PI3K)/AKT/mTOR, Phospholipase C (PLC)/ Protein kinase B (PKC), Mitogen-activated protein kinase (MAPK) family, pyk2 and several other molecules [34,47,48]. It has also been reported that SP1 could facilitate CCR7 function by binding to specific sites in its promoter region [49,50]. Additionally, in HNSCC cell lines with high invasiveness (PCI-4B/37B) miR-92b and CCR7 expression were both elevated compared with in PCI-4A/37A cells. We suggest that the SP1/miR-92b regulatory loop may promote HNSCC metastasis via CCR7 signaling. This is also what we need to study in the future.

## Conclusion

In conclusion, in this study we identified an SP1/miR-92b feedback loop that could promote HNSCC cell invasion and metastasis. Our data suggested the oncogenic role of transcriptional factors SP1 and miR-92b, and highlighted a potential treatment targeting SP1/miR-92b in head and neck squamous cell carcinoma patients.

## Supplementary Material

Supplemental Material

## Data Availability

The data used to support the findings of this study are available from the corresponding author upon request. Raw data of the nude mice tumor growth, Real-time qPCR and Western blot was shown in Tables S3, S4 and Supplementary material 4, respectively.
