# Supplementary material for: Specificity protein 1/microRNA-92b forms a feedback loop promoting the migration and invasion of head and neck squamous cell carcinoma
Source: Bioengineered. 2021 Dec 14;12(2):11397–409. doi: 10.1080/21655979.2021.2008698 (PMC8810166; doi:10.1080/21655979.2021.2008698)
Supplement: Supplemental Material [file KBIE_A_2008698_SM0937.zip › supplementary/Supplementary material S1.docx]

## Supplementary material S1. Microarray Methods

miRNA microarray

The 7th generation of miRCURY^TM^ LNA Array (v.18.0) (Exiqon) contains 3100 capture probes, covering all human, mouse and rat microRNAs annotated in miRBase 18.0, as well as all viral microRNAs related to these species. In addition, this array contains capture probes for 25 miRPlus™ human microRNAs.

RNA extraction

Total RNA was isolated using TRIzol (Invitrogen) and miRNeasy mini kit (QIAGEN) according to manufacturer’s instructions, which efficiently recovered all RNA species, including miRNAs. RNA quality and quantity was measured by using nanodrop spectrophotometer (ND-1000, Nanodrop Technologies) and RNA Integrity was determined by gel electrophoresis.

RNA labeling

After RNA isolation from the samples, the miRCURY™ Hy3™/Hy5™ Power labeling kit (Exiqon, Vedbaek, Denmark) was used according to the manufacturer’s guideline for miRNA labelling. One microgram of each sample was 3'-end-labeled with Hy3^TM^ fluorescent label, using T4 RNA ligase by the following procedure: RNA in 2.0 μL of water was combined with 1.0 μL of CIP buffer and CIP (Exiqon). The mixture was incubated for 30 min at 37°C, and was terminated by incubation for 5 min at 95°C. Then 3.0 μL of labeling buffer, 1.5 μL of fluorescent label (Hy3^TM^), 2.0 μL of DMSO, 2.0 μL of labeling enzyme were added into the mixture. The labeling reaction was incubated for 1 h at 16°C, and terminated by incubation for 15 min at 65°C.

Array hybridization

After stopping the labeling procedure, the Hy3^TM^-labeled samples were hybridized on the miRCURY^TM^ LNA Array (v.18.0) (Exiqon) according to array manual. The total 25 μL mixture from Hy3^TM^-labeled samples with 25 μL hybridization buffer were first denatured for 2 min at 95°C, incubated on ice for 2 min and then hybridized to the microarray for 16–20 h at 56°C in a 12-Bay Hybridization Systems (Hybridization System - Nimblegen Systems, Inc., Madison, WI, USA), which provides an active mixing action and constant incubation temperature to improve hybridization uniformity and enhance signal. Following hybridization, the slides were achieved, washed several times using Wash buffer kit (Exiqon), and finally dried by centrifugation for 5 min at 400 rpm. Then the slides were scanned using the Axon GenePix 4000B microarray scanner (Axon Instruments, Foster City, CA).

Data analysis

Scanned images were then imported into GenePix Pro 6.0 software (Axon) for grid alignment and data extraction. Replicated miRNAs were averaged and miRNAs that intensities>=30 in all samples were chosen for calculating normalization factor. Expressed data were normalized using the Median normalization. After normalization, differentially expressed miRNAs were identified through Fold Change filtering. Hierarchical clustering was performed using MEV software (v4.6, TIGR).
