# Supplementary material for: Specificity protein 1/microRNA-92b forms a feedback loop promoting the migration and invasion of head and neck squamous cell carcinoma
Source: Bioengineered. 2021 Dec 14;12(2):11397–409. doi: 10.1080/21655979.2021.2008698 (PMC8810166; doi:10.1080/21655979.2021.2008698)
Supplement: Supplemental Material [file KBIE_A_2008698_SM0937.zip › supplementary/Supplementary material S2.docx]

## Supplementary material S2. Analysis of cell proliferation, migration and invasion

Migration and invasion assays were performed using Transwell chambers (3422, 24-well insert; 8-μm pore size; Corning Costar, NY, USA) coated with or without Matrigel (356234, BD Biosciences, NJ, USA). 4 × 10^5^ HNSCC cells in DMED medium were placed into the upper chamber, and medium supplementary with 10 % FBS was added to the lower chamber. Then PCI-37A cells were incubated for 24 hours for migration and 48 hours for invasion, and PCI-37A cells were incubated for 8 hours for migration and 24 hours for invasion. Then the migratory or invasive cells were fixed by 4% paraformaldehyde, stained with 0.1% crystal violet, and counted in three random fields under 200x magnification.

For monolayer wound healing assay, cells grew in 6-well plates in DMEM with a confluence at 90% were scrapped with a pipette tip to create a cell-free area. Then each group was immediately transfected with corresponding RNA oligos. The wound healing status was captured at 0h, 24h and 48h post-transfection at 100x magnifications.

CCK-8 cell proliferation assay was performed in 96-well plates with 5,000 transfected cells per well. Each groups were cultured until 24, 48 and 72 hours after transfection, then each well was added with 10uL CCK-8 reagent (CK04, Dojindo, MD, USA) and incubated for another 2 hours at 37℃. At the end of incubation the number of viable cells were evaluated by measurement of the optical density value (OD value) at 450 nm. Each cell functional test was measured in triplicates.
