# Supplementary material for: Specificity protein 1/microRNA-92b forms a feedback loop promoting the migration and invasion of head and neck squamous cell carcinoma
Source: Bioengineered. 2021 Dec 14;12(2):11397–409. doi: 10.1080/21655979.2021.2008698 (PMC8810166; doi:10.1080/21655979.2021.2008698)
Supplement: Supplemental Material [file KBIE_A_2008698_SM0937.zip › supplementary/Supplementary material S3.docx]

## Supplementary material S3. ChIP assay experimental groups

The binding of each predicted sites were evaluated with 7 parallel groups:

(A) experiment group: immunoprecipitated with SP1 antibody and amplified with targeted primers;

(B) RT-Input1: without immunoprecipitation and amplified with targeted primers;

(C) negative control group: immunoprecipitated with host IgG and amplified with targeted primers;

(D) PCR negative control: using ddH_2_O as template and amplified with targeted primers;

(E) positive control group: immunoprecipitated with RNA polymerase II and amplified with GAPDH primers;

(F) RT-Input2: without immunoprecipitation and amplified with GAPDH primers;

(G) WB-Input: western bloting SP1 protein expression in total DNA-protein crosslink.
