# Supplementary material for: Specificity protein 1/microRNA-92b forms a feedback loop promoting the migration and invasion of head and neck squamous cell carcinoma
Source: Bioengineered. 2021 Dec 14;12(2):11397–409. doi: 10.1080/21655979.2021.2008698 (PMC8810166; doi:10.1080/21655979.2021.2008698)
Supplement: Supplemental Material [file KBIE_A_2008698_SM0937.zip › supplementary/Table S2.docx]

| Table S3. HNSCC patients information | | | | |
| --- | --- | --- | --- | --- |
| Patient number | Gender | Age | Tumor location | TNM stage |
| 1 | M | 59 | Buccal mucosa | T3N0M0 |
| 2 | M | 54 | Tongue and soft palate | T1N0M0 |
| 3 | F | 64 | Buccal mucosa | T3N0M0 |
| 4 | M | 50 | Tongue | T2N1M0 |
| 5 | F | 66 | Buccal mucosa | T1N1M0 |
| 6 | F | 54 | Tongue | T2N0M0 |
| 7 | M | 51 | Floor of mouth | T1N2M0 |
| 8 | M | 47 | Soft palate and parapharyngeal mucosa | T2N0M0 |
| 9 | M | 68 | Tongue | T2N0M0 |
| 10 | M | 61 | Lower gums | T2N2M0 |
| 11 | M | 72 | Tongue | T2N0M0 |
| 12 | M | 63 | Tongue | T3N2M0 |
| 13 | F | 61 | Tongue | T2N0M0 |
| 14 | M | 58 | Tongue | T2N0M0 |
| 15 | M | 50 | Tongue | T2N0M0 |
