# Supplementary figures and images for: Specificity protein 1/microRNA-92b forms a feedback loop promoting the migration and invasion of head and neck squamous cell carcinoma
Source: Bioengineered. 2021 Dec 14;12(2):11397–409. doi: 10.1080/21655979.2021.2008698 (PMC8810166; doi:10.1080/21655979.2021.2008698)

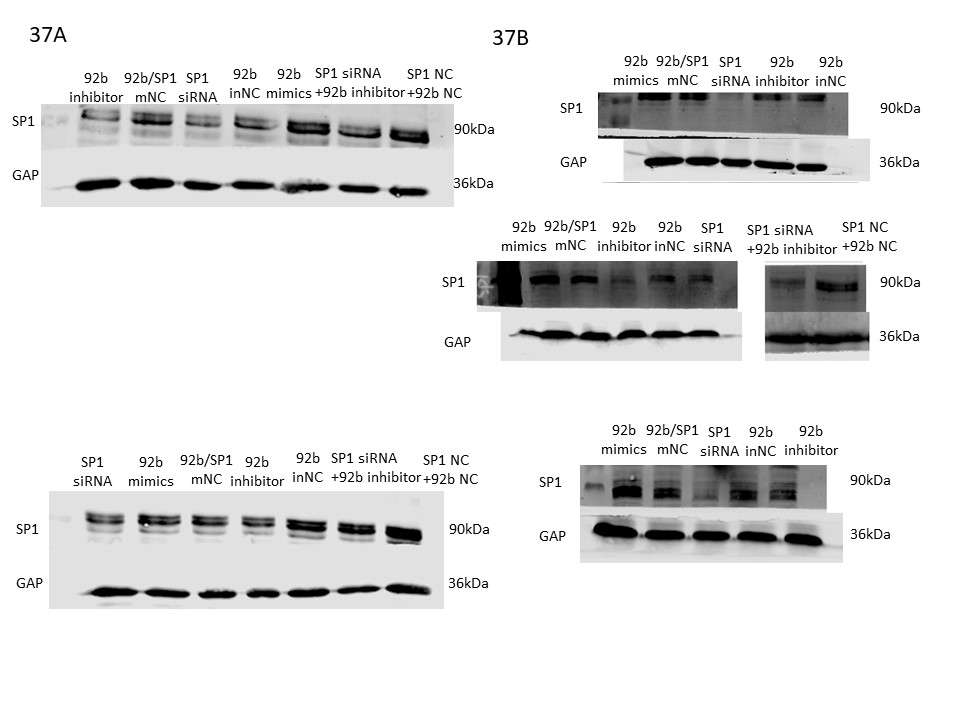

Supplement: Supplemental Material [file KBIE_A_2008698_SM0937.zip › supplementary/Supplementary material S4.JPG]
